# Predictors of Recurrent Sickness Absence Due to Depressive Disorders – A Delphi Approach Involving Scientists and Physicians

**DOI:** 10.1371/journal.pone.0051792

**Published:** 2012-12-20

**Authors:** Giny Norder, Corné A. M. Roelen, Willem van Rhenen, Jan Buitenhuis, Ute Bültmann, Johannes R. Anema

**Affiliations:** 1 365/Occupational Health Service Utrecht, Utrecht, The Netherlands; 2 Department of Health Sciences, University Medical Center Groningen, University of Groningen, Groningen, The Netherlands; 3 Center for Human Resource, Organization and Management Effectiveness, Business University Nyenrode, Breukelen, The Netherlands; 4 Univé Insurances Medical Department, Assen, The Netherlands; 5 Department of Public and Occupational Health, EMGO+ Institute for Health and Care Research, VU University Medical Center, Amsterdam, The Netherlands; 6 Research Center for Insurance Medicine, Amsterdam, The Netherlands; Catholic University of Sacred Heart of Rome, Italy

## Abstract

**Background:**

Depression is a common and highly recurrent mental disorder that is accompanied by poor functioning at home and at work. Not all depressed employees report sick and little is known about variables associated with sickness absence (SA) due to depression. Recurrent SA due to depression tends to marginalize employees from the workforce and exclude them from social participation. Therefore, this study sought group consensus on factors predicting recurrent SA due to depression.

**Methodology/principal findings:**

23 scientists in the field of work and mental health and 23 physicians with expertise in assessing work disability were invited for a Delphi study. Sixty-seven factors retrieved from the literature were scored for their impact on the recurrence of SA due to depression, range 1 (no impact) to 10 (very high impact) in two Delphi rounds. The third Delphi round addressed the assessability and modifiability of elected predictors. Group consensus was defined as 75% agreement. In the first round (response 78%), group consensus was reached on a high impact of 13 factors on recurrent SA due to depression. The second round (response 79%) added another 8 factors with high impact on recurrent SA due to depression. The panelists were of the opinion that stressful life and work events, age at first diagnosis, duration of the last depressive episode, anxiety, lifetime number of depressive episodes, and psychological work demands were readily assessable in consultation with patients. Furthermore, work factors, particularly decision latitude, psychological job demands, and commitment to work, were recognized as modifiable.

**Conclusions/significance:**

Although results have to be validated with further quantitative research, physicians may identify employees at risk of recurrent SA due to depression and may support them to adjust their work aimed at increasing commitment to work and preventing future SA due to depression.

## Introduction

Depression is a common disorder in developed countries with a lifetime prevalence of up to 25% for women and 12% for men [1]. Depression is characterized by substantial impairment of an individual's ability to take care of everyday responsibilities at home and at work [2]. Depressed persons are more often unemployed or on sickness absence (SA), and have more work performance deficits than non-depressed persons [3], [4]. Studies of work productivity and functioning of depressed employees most frequently consider disease-related factors, whereas personal factors and work-related factors are less frequently addressed. Work productivity was found to be strongly associated with the duration of depression and moderately with the severity of depression, co-morbid mental or physical disorders, older age, and a history of previous SA or work disability [5].

After recovery from an episode of depression, the chances of maintaining that recovery decrease over time [1]. Theory and research have assumed a chronic and recurrent disease model for depression [6]. In a systematic review of the literature, Hardeveld et al. (2010) reported recurrences in 85% of patients in specialized mental healthcare settings and in 35% of persons in the general population [7]. Since 2000, literature reviews have reported a total of 67 factors predicting recurrences of depression [7]–[16].

Factors associated with recurrent depression may differ from those associated with recurrent SA due to depression, as depression not always results in SA. Some depressed employees may report sick, while others stay at work. In a Norwegian population survey, the number of men and women who had consulted a physician or taken medication because of mental health problems were 11 and 18 times higher, respectively, than the number of men and women who had SA due to mental health problems [17]. If depressed individuals report sick, they often stay off work for a long period of time and may even transfer to disability pension after one year of SA [18]–[23], explaining part of the economic burden and high societal costs of depression [24]–[26].

Long-term SA, especially recurrent long-term SA, increases the probability of being excluded from the workforce and consequently threatens social participation. Therefore, it is important to identify employees at risk of recurrent SA due to depression. High-risk employees can then be invited for counselling and, if appropriate, referred to targeted interventions [27], all the more because the course of depression worsens with each recurrence [1]. Systematic reviews of the literature have revealed predictors of recurrent depression, but there is no literature on predictors of recurrent SA due to depression. The purpose of this study was to reach group consensus on a set of predictors of recurrent SA due to depression by using a Delphi approach.

## Methods

### Study design

The Delphi approach is an iterative multistage process designed to transform personal opinion into group consensus [28]. A modified Delphi procedure consisting of several rounds was used to reach consensus on predictors of recurrent SA due to depression. The first Delphi round took place in a period of four months directly followed by a second Delphi round which took three months. A third Delphi round was used to determine whether or not the elected predictors were assessable and modifiable.

### Ethics statement

The study was approved by the Medical Ethics Committee of the University Medical Center Groningen, who advised us that written informed consent was not necessary because participants in the Delphi procedure were neither subject to treatments nor engaged in specific behaviors. Participants provided verbal informed consent to participate in the Delphi procedure by e-mail. This consent procedure was approved by the Medical Ethics Committee of the University Medical Center Groningen.

### Study definitions

#### Depression

Depression is a mood disturbance characterized by a loss of interest or pleasure in normal everyday activities. Depression was defined according to the criteria of the International Classification of Diseases (ICD-10) and encompassed depressive episodes (ICD-10 F32), recurrent depressive disorders (ICD-10 F33), and persistent mood disorders (ICD-10 F34) such as dysthymia. Depressive symptoms within two months of the loss of a loved one were not included because depression cannot be diagnosed if depressive symptoms are better accounted for by bereavement [29]. Furthermore, bipolar depressive disorders (ICD-10 F31) were not included in the definition of depression, because bipolar depressive disorders share more risk factors, neural substrates, cognitions and endophenotypes with schizophrenia than with unipolar depression [30].

#### Sickness absence

Sickness absence (SA) was defined as a financially compensated temporary medically certified absence or leave from work, due to any (i.e., work-related as well as non work-related) illness or injury. In the Delphi procedure, the focus was on recurrent long-term SA due to depression. Long-term SA is defined differentially across countries. In this study, we averaged the duration of long-term SA to 2 months in line with DSM-IV criteria, stating that depressive disorder can be diagnosed if symptoms persist for longer than 2 months [29]. Long-term SA was maximized at 1 year because SA compensation is restricted to a 1-year period in many countries, after which employees are transferred to other types of compensation such as rehabilitation allowances or disability pensions.

A recurrence was defined as a new SA episode due to depression occurring ≥4 weeks after a previous SA episode as Dutch sickness insurance policies regard two SA episodes with less than 4 weeks worked between them as one SA period. Koopmans et al. demonstrated that 90% of recurrences of mental SA occurred within 3 years of a first mental SA episode [31]. Therefore, we instructed the Delphi panelists that recurrences were assumed to occur within 3 years of recovery from a previous SA episode due to depression.

### Panelists

Purposive convenience sampling was used to construct a panel of 46 members with scientific (n = 23) or professional (n = 23) expertise in the field of mental health and work. Twenty scientists, regarded by the authors as experts in the field of mental health and work, were contacted via e-mail to ask whether they: i) had the expertise and time to participate in the Delphi procedure, and ii) knew scientists in the field that had yet to be contacted. Thirteen scientists agreed to participate and they suggested another 10 scientists. Hence, a total of 23 scientists of whom 8 were from the Netherlands, 7 from the rest of Europe, and 8 from North America participated in the Delphi procedure. At inclusion, the scientists had been publishing on mental health and work issues in international peer-reviewed journals abstracted in Medline for on average 13 years (range 2–27 years). To add opinions from the work field, occupational physicians working at 365/ArboNed and physicians of the Dutch Association of Medical Insurance Advisors were invited to participate in the Delphi procedure. A total of 23 physicians with an experience in the assessment of work disability for on average 19 years (range 11–33 years) agreed to participate in the Delphi procedure.

### Data collection

A total of 67 factors associated with recurrent depression were retrieved from recent literature reviews [7]–[15] and the Dutch multidisciplinary guideline for depression [16]. To assess their impact on recurrent SA due to depression, a score range of 1 (no impact) to 10 (very high impact) was applied to each of these factors. Subsequently, the factors were categorized as person-related factors, disease-related factors, and work-related factors. Person-related factors included sociodemographics (e.g., age, gender, socioeconomic and marital status, number of children living at home, and care for others), family history (e.g. depression of parents or other family members and childhood life events or adversities), and cognitions (e.g., neuroticism, irrational beliefs, self-efficacy, and self-esteem). Examples of disease-related factors were the severity and duration of depression, comorbid psychopathology, and both work and social dysfunctioning as a result of depression. Work-related factors involved exposures to noise, light, or toxics as well as factors such as job demands, job control, work efforts and rewards, commitment to work, support at the workplace, and stressful evens/bullying at work. Apart from an opinion on the impact of each separate factor, an overall opinion was asked on the impact of these categories with a score range of 1 (no impact) to 10 (very high impact). The Delphi questionnaire was sent by e-mail to the Delphi panelists and reminders were sent monthly.

After the first round, the predictors on which consensus was reached were removed from the Delphi questionnaire. Hence, the second round Delphi questionnaire only included the factors on which no consensus was reached yet, together with their first round score range to provide the panellists with some feedback [28]. The second round Delphi questionnaire was sent by e-mail to the panelists and reminders were sent monthly.

In the third Delphi round, the panelists were asked to indicate if the elected predictors are readily assessable by physicians in consultation with patients. The panelists were also asked to give their opinion on the modifiability of the elected predictors by asking them how much effort would be required to change a predictor [32].

### Data analysis

As there is no universal agreement for Delphi procedures, consideration must be given to the level of consensus. McKenna (1994) suggested that consensus should be equated with 51% agreement amongst respondents [33], whereas Sumsion (1998) recommended 70% agreement [34] and Green et al. (1999) proposed 80% agreement [35]. In this study, group consensus was defined as a 75% agreement. If 75% or more of the panelists rated a factor with a score ≥7 (on a range of 1–10), this was considered as group consensus on high impact on recurrent SA due to depression. Furthermore, if 75% of the panelists regarded a factor as assessable in consultation with employees, this was considered as group consensus that the factor was readily assessable and if 75% of the panelists thought that predictors could be modified with little or some effort, this was regarded as group consensus on the modifiability of predictors.

Differences between the rating of scientists and physicians were examined with the non-parametric Mann-Whitney and Fischer's exact tests in SPSS for Windows, version 20. Statistical significance was concluded for p<0.05.

## Results

### First Delphi round

In the first round, 36 panel members (78%) returned the Delphi questionnaire, of which 18 were scientists and 18 physicians. Three panel members (2 scientists in the field of sociology and 1 physician) did not complete the questionnaire, because on second thought they considered themselves not knowledgeable on the subject. The remaining 16 scientists had a higher score on the category of work-related predictors than 17 physicians (Table 1), meaning that scientists accredited work factors a higher impact on recurring SA due to depressive disorders. Physicians accredited more impact to person-related predictors, particularly sociodemographic variables and family history.

**Table 1:** Categories for predicting recurrent sickness absence due to depression.

	Scientists	Physicians	Mann-Whitney
Person-related factors			
Sociodemographics	5 (5–7)	7 (6–8)	P<0.01
Family history	4 (3–6)	5 (5–7)	P = 0.0
Personality and cognitions	6.5 (5–7)	7 (6–7)	P = 0.82
Disease-related factors	8 (7–9)	8 (7–9)	P = 0.97
Work-related factors	7 (6–9)	6 (5–7)	P = 0.02

The table shows median scores (interquartile range) of 16 scientists with a 13-year experience in the field of mental health and work and 17 physicians with a 19-year experience in assessing work disability due to mental health problems, on a range from 1 to 10.

Group consensus was reached on a high impact of the lifetime number of depressive episodes, substance abuse, residual symptoms after resuming work, social and work dysfunctioning, comorbid anxiety and comorbid DSM (axis I and II) disorders, neuroticism, stressful events in private life or at work, commitment to work, and high demands in work combined with low control over work (Table 2).

**Table 2:** Results of Delphi rounds 1 and 2.

	Round 1			Round 2		
	Scientists	Physicians	Total	Scientists	Physicians	Total
Lifetime number of episodes^a^	88%	100%	**94%**			
Substance abuse	75%	100%	**88%**			
Work dysfunctioning	94%	82%	**88%**			
Social dysfunctioning	75%	82%	**85%**			
DSM axis II personality disorders	75%	88%	**82%**			
High demands – low control	81%	82%	**82%**			
Stressful work events	81%	82%	**82%**			
Residual symptoms	69%	88%	**79%**			
DSM axis I psychopathology	75%	82%	**79%**			
Anxiety	75%	82%	**79%**			
Neuroticism	76%	76%	**76%**			
Stressful life events	75%	76%	**76%**			
Commitment to work	75%	76%	**76%**			
Severity of first episode^a^	50%	65%	58%	93%	84%	**88%**
Severity of last episode^a^	75%	65%	70%	93%	79%	**85%**
Duration of last episode^a^	63%	76%	70%	93%	68%	**79%**
Duration of first episode^a^	44%	65%	55%	80%	79%	**79%**
Effort – reward imbalance	69%	59%	64%	80%	79%	**79%**
Age at first episode^a^	50%	82%	67%	73%	84%	**79%**
Decision latitude	75%	53%	64%	80%	74%	**76%**
Psychological job demands	88%	59%	73%	80%	70%	**75%**
Co-worker support	63%	71%	67%	73%	74%	74%
Supervisor support	75%	65%	70%	80%	63%	71%
Pessimism	63%	65%	64%	60%	79%	71%
Bullying at workplace	88%	47%	67%	87%	53%	68%
Role conflicts in work	56%	47%	52%	67%	68%	68%
Self-esteem	56%	53%	55%	53%	74%	65%
Self-efficacy	50%	41%	45%	53%	74%	65%
Job insecurity	44%	65%	55%	73%	58%	65%
Social support	38%	71%	55%	40%	79%	62%

The table shows the 30 highest scoring factors and the percentages of panelists who scored the factor ≥7 (i.e. high impact on recurrent sickness absence due to depression); bold font indicates consensus.

a episode of depression.

### Second Delphi round

In the second Delphi round, 34 (79%) of 43 remaining panel members returned the questionnaire and group consensus was reached on another 8 predictors: age at the time of the first depressive episode, severity and duration of the first and last depressive episodes, and the work factors psychological demands, effort-reward imbalance, and decision latitude (Table 2).

### Third Delphi round

In the third Delphi round, the panelists gave their opinion on whether or not the high-impact factors were readily assessable and modifiable. A total of 36 panelists (81%) returned the questionnaire and reached group consensus that stressful life and work events, age at first diagnosis, duration of the last depressive episode, anxiety symptoms, the lifetime number of depressive episodes, and psychological work demands were readily assessable in consultation with patients (Table 3). Scientists and physicians did not differ significantly in their opinion on the assessability of predictors.

**Table 3:** Delphi round 3: Is the elected factor assessable in consultation with the patient?

	Total	Don't		Scientists	Physicians	Fisher's exact test
	Yes (%)	No	know	Yes (%)	Yes (%)	
**Stressful life events**	**33 (94)**	2	0	17 (94)	16 (94)	P = 0.743
**Age at first diagnosis**	**32 (91)**	2	1	15 (83)	17 (100)	P = 0.242
**Duration last episode**	**31 (89)**	4	0	16 (89)	15 (88)	P = 0.677
**Anxiety**	**30 (86)**	2	3	16 (89)	14 (83)	P = 0.726
**Stressful work events**	**28 (80)**	4	3	17 (94)	11 (63)	P = 0.210
**Lifetime episodes**	**27 (77)**	7	1	12 (67)	15 (88)	P = 0.199
**Psychological demands**	**27 (77)**	7	1	14 (78)	13 (76)	P = 0.500
Decision latitude	26 (74)	3	6	14 (78)	12 (71)	P = 0.580
Severity last episode	26 (74)	5	5	12 (67)	14 (83)	P = 0.186
Substance abuse	25 (71)	6	4	15 (83)	10 (59)	P = 0.072
Commitment to work	24 (69)	7	3	12 (67)	12 (71)	P = 0.539
Duration first episode	22 (63)	10	3	11 (61)	11 (65)	P = 0.445
Work dysfunctioning	22 (63)	10	3	12 (67)	10 (59)	P = 0.541
Residual symptoms	20 (57)	10	5	13 (72)	7 (41)	P = 0.181
High demands low control	20 (57)	12	3	9 (50)	11 (65)	P = 0.358
DSM axis I pathology	19 (54)	12	4	7 (39)	12 (71)	P = 0.033
Social dysfunctioning	19 (54)	12	4	11 (61)	8 (47)	P = 0.638
Effort-reward imbalance	19 (54)	13	3	10 (56)	9 (53)	P = 0.615
Neuroticism	18 (51)	11	6	7 (39)	11 (65)	P = 0.082
Severity first episode	12 (34)	15	8	7 (39)	5 (29)	P = 0.552
DSM axis II pathology	9 (26)	21	5	5 (28)	4 (24)	P = 0.596

The table shows the number of panelists per answer category and Fisher's exact test of differences between scientists and physicians; bold font represents consensus.

Although both scientists and physicians indicated that work factors, particularly decision latitude, psychological job demands, and commitment to work, were best modifiable, no group consensus was reached on the modifiability of predictors (Table 4).

**Table 4:** Delphi round 3: How much effort is needed to change the elected factor?

	Don't know	Yes, with little or some effort	Not easily modifiable
Decision latitude	4	17 (55%)	14 (45%)
Psychological demands	2	17 (52%)	16 (48%)
Commitment to work	3	14 (44%)	18 (56%)
Social dysfunctioning	1	16 (47%)	18 (53%)
Work dysfunctioning	1	16 (47%)	18 (53%)
Anxiety	4	14 (45%)	17 (55%)
Effort – reward imbalance	2	15 (45%)	18 (55%)
DSM-axis I pathology	8	11 (41%)	16 (59%)
Stressful work events	3	13 (41%)	19 (59%)
High demands low control	2	13 (39%)	20 (61%)
Residual symptoms	5	11 (37%)	19 (63%)
Substance abuse	1	8 (24%)	26 (76%)
Duration last episode	2	4 (12%)	29 (88%)
Neuroticism	4	3 (10%)	28 (90%)
Severity last episode	3	3 (9%)	29 (91%)
Lifetime episodes	-	3 (9%)	32 (91%)
Stressful life events	2	2 (6%)	31 (94%)
Severity first episode	1	2 (6%)	32 (94%)
Duration first episode	1	1 (3%)	33 (97%)
DSM-axis II pathology	5	0	30 (100%)
Age at first diagnosis	3	0	32 (100%)

The table shows the number (%) of panelists per answer category.

## Discussion

Of 67 factors reported in the literature to predict recurrences of depression, 21 were thought important for predicting recurrent sickness absence (SA) due to depression. Scientists and physicians reached consensus on a high impact of predictors associated with the clinical picture, though these predictors were estimated as difficultly or not modifiable. Work factors, especially decision latitude, psychological job demands, and commitment to work were thought to be best modifiable, although no group consensus was reached on the modifiability of variables predicting recurrent SA due to depression.

### Strengths and weaknesses

An asset of the study was the high response rate in all three Delphi-rounds. A response rate >70% was suggested by Sumsion (1998) to be essential to maintain the rigor of the Delphi approach [34]. Furthermore, the Delphi approach combined the opinions of experts and professionals in the field of mental health and work into group consensus.

Green et al. (1999) have shown that two or three rounds sufficed to reach consensus [35]. The present study used two rounds during which no items were added and the wording of items remained exactly the same [28]. In the second Delphi round, the panelists were informed about the first round score ranges of items, which enabled them to see where their responses stood in relation to the group. Such feedback contributes to reaching group consensus, though it was not possible to find out how much the panelists relied on it [28].

The panelists did not meet with each other face to face, which enabled them to react unbiased of the identities and pressures of others [33], [36]. A disadvantage may be that consensus was weakened by not allowing panelists to discuss the issues raised and elaborate on their views [36], [37].

Although the Delphi approach is based upon the assumption of safety in numbers, meaning that several people are less likely to arrive at a wrong decision than a single individual, there is no evidence of the reliability of the Delphi method in terms of reproducibility [28], [36], [37]. In other words: if the same information was given to other panels, the results may not necessarily be the same. The drawing of a convenience sample may have further undermined the Delphi's forecasting ability, though this weakness was partially dealt with by asking the panelists for more experts or professionals from their networks.

With regard the validity of the Delphi procedure, the use of experts on mental health and work, and professionals with experience in the assessment of work disability of depressed employees helped to increase content validity and the use of successive rounds with the same questionnaire helped to increase the concurrent validity [37].

### Predictors of recurrent sickness absence due to depression

Of the 21 factors with assumed high impact on recurrent SA due to depression, 11 (52%) were associated with the clinical picture of depression. Obviously, the panelists accredited most importance to the clinical picture for predicting recurrent SA due to depression. Although in line with earlier findings [7], [38], the results may have been biased by existing literature, which most often reports on the relationship between disease-related factors and both work productivity of depressed employees [5].

Scientists valued work factors as more important predictors of recurrent SA due to depression than physicians did. Possibly, physicians have a more ‘clinical look’ and pay attention to the medical aspects of SA, whereas scientists may consider SA from a more environmental context. Irrespective of the different appraisal of work factors, both scientists and physicians were of the opinion that work factors were best modifiable. Hence, interventions to prevent recurrent SA due to depression could be targeted at work conditions, especially decision latitude in work, psychological job demands, and commitment to work. In this regard, it is interesting to note that participatory workplace interventions, consisting of a stepwise process to support employees and supervisors in identifying and solving obstacles for return to work, was effective for sustainable return to work of sick-listed employees with distress [39]. Participatory workplace interventions may offer an opportunity for depressed employees and their supervisors to discuss and adjust barriers in work to prevent recurrent SA due to depression.

The current study sought for group consensus among scientists and physicians. Other stakeholders such as supervisors and employees were not involved in this Delphi study. Earlier research has shown that employees, supervisors and occupational physicians differ in their opinion in what they see as important for return to work after SA due to depression [40]. The perspectives of physicians and supervisors were generally more similar to each other than to employees' perspectives. Employees' perspectives were not included in this study because these are less important for the predictability of recurrent SA since most individuals will not anticipate future illnesses or injuries.

### Meaning of the study

From systematic reviews of the literature in the last decade, a total of 67 variables were found to be associated with recurrent depression. Not all depressed patients will report sick, i.e. recurrent depression is not the same as recurrent SA due to depression. SA has important societal, organizational and personal consequences. For example, SA excludes individuals from work and marginalizes their social participation. Besides, SA is a strong predictor of future poor health, low mental well-being, low work ability and increased mortality [41]–[44]. During a 13-year follow-up, the hazard ratio for mortality was 1.9 for SA with psychiatric diagnoses [41]. Furthermore, psychiatric and non-psychiatric SA predict future depression with a fully adjusted odds ratio of 1.53 for one SA episode and 1.95 for two SA episodes [45]. Obviously, it is important to identify which depressed patients are at risk of SA, especially recurrent SA. By using a Delphi approach, expert group consensus was reached that stressful life and work events, age at first diagnosis, duration of the last depressive episode, anxiety symptoms, the lifetime number of depressive episodes, and psychological work demands are predictors of recurrent SA due to depression that are readily assessable in consultation with patients without the use of questionnaires or other diagnostic tools. Although these 7 variables have yet to be further validated in quantitative research, physicians may use them to decide which depressed employees should be followed and monitored because of their risk of recurrent SA. Physicians may decide to counsel employees at risk of recurrent SA due to depression to evaluate their symptoms and needs, advise work adjustments or refer them to specialist treatment in order to prevent recurrent SA.
